# CoPAP: Coevolution of Presence–Absence Patterns

**DOI:** 10.1093/nar/gkt471

**Published:** 2013-06-08

**Authors:** Ofir Cohen, Haim Ashkenazy, Eli Levy Karin, David Burstein, Tal Pupko

**Affiliations:** Department of Cell Research and Immunology, George S. Wise Faculty of Life Sciences, Tel Aviv University, Ramat Aviv 69978, Israel

## Abstract

Evolutionary analysis of phyletic patterns (phylogenetic profiles) is widely used in biology, representing presence or absence of characters such as genes, restriction sites, introns, indels and methylation sites. The phyletic pattern observed in extant genomes is the result of ancestral gain and loss events along the phylogenetic tree. Here we present CoPAP (coevolution of presence–absence patterns), a user-friendly web server, which performs accurate inference of coevolving characters as manifested by co-occurring gains and losses. CoPAP uses state-of-the-art probabilistic methodologies to infer coevolution and allows for advanced network analysis and visualization. We developed a platform for comparing different algorithms that detect coevolution, which includes simulated data with pairs of coevolving sites and independent sites. Using these simulated data we demonstrate that CoPAP performance is higher than alternative methods. We exemplify CoPAP utility by analyzing coevolution among thousands of bacterial genes across 681 genomes. Clusters of coevolving genes that were detected using our method largely coincide with known biosynthesis pathways and cellular modules, thus exhibiting the capability of CoPAP to infer biologically meaningful interactions. CoPAP is freely available for use at http://copap.tau.ac.il/.

## INTRODUCTION

A phyletic pattern (also termed phylogenetic profile) is a binary-coded data set in which presence (‘1’) versus absence (‘0’) of homologous characters is denoted across species. This 0/1 matrix is equivalent to a gap-free multiple sequence alignment, in which rows correspond to species and columns correspond to binary characters. Phyletic pattern representation is useful for evolutionary analysis of various types of data including gene families (1–3), restriction sites (4–6), indels (7,8), introns (9,10) and morphological characters [reviewed in (11)].

Methods for evolutionary analysis of phyletic patterns have progressed from the traditional parsimony (1) to likelihood models, in which the dynamics of gain (0→1) and loss (1→0) events are assumed to follow a continuous-time Markov process (9,10,12,13). Recently, we have implemented a stochastic-mapping approach that uses advanced evolutionary mixture models to accurately infer branch-site specific events (14). We have shown that our stochastic-mapping approach is over two folds more accurate in detecting branch-specific events compared with the prevalent maximum-parsimony approach (15).

Previous studies have shown that genomes evolve under various constraints, which are reflected in correlated evolutionary histories. Examples include coevolving sites within a protein (16–18) and coevolutionary interactions between different genes (19–27). Importantly, many of these studies have demonstrated that coevolutionary interactions between genes are highly suggestive of functional interactions [reviewed in (28)]. In the case of prokaryotic genomes, coevolutionary interactions between genes can be inferred from phyletic patterns by searching for co-occurrence of gene gain (resulting from horizontal gene transfer) and loss events. Several evolutionary methods to infer coevolutionary interactions from phyletic patterns exist, ranging from maximum-parsimony methods (29,30) to methods that provide explicit models of coevolution (31). Recently, we developed a probabilistic method to infer coevolutionary interactions from phyletic patterns (32). In contrast to the maximum-parsimony approach, our method heavily relies on advanced probabilistic models for mapping gain and loss events along the tree. Moreover, unlike explicit models for pairwise coevolution (31), our method allows analyzing data sets with thousands of characters and hundreds of species.

Here we present CoPAP (Coevolution of Presence–Absence Patterns), a user-friendly web server which is the first publically available web server for coevolutionary analysis of phyletic data. The main features and novelties of our web server are as follows: (i) usage of efficient probabilistic methods, capable of analyzing evolutionary interactions across hundreds of genomes (see case study below); (ii) implementation of various evolutionary models including complex mixture models, which can accurately capture gain–loss dynamics; (iii) visualization and analysis of the inferred coevolutionary network using Cytoscape (33) with additional preloaded plug-ins to study clusters within the network (34); (iv) providing benchmark data sets of both coevolving and independently evolving genes; (v) phylogenetic visualization of the phyletic patterns using tree visualization applets; (vi) multiple advanced options for expert users, while providing novice users with a minimalistic interface, which enables fast and reliable results for typical inputs.

## RESULTS

### Input

The CoPAP input is a phyletic pattern provided as a 0/1 matrix. A phylogenetic tree is either provided as input by the user or estimated from the phyletic pattern by the neighbor joining (NJ) algorithm (35). For NJ, distances among genomes are computed using maximum likelihood (a two state model, in which the stationary frequencies are estimated by counting). CoPAP allows for an optional input with description and annotation of characters (e.g. gene information) to facilitate biological interpretation of the resulting coevolutionary network. While the method is suitable for analyzing various types of binary data, we will refer to genes throughout the manuscript to facilitate readability. We note that CoPAP can only analyze binary characters, and therefore cannot capture evolutionary events such as variation in gene copy number [see for example (29)].

### Coevolution computation

CoPAP infers coevolutionary interactions and computes statistical significance using simulations. For methodological details see (32) as well as the ‘Overview’ section in the CoPAP web server. Parameters that can be adjusted by the user include, for example, controlling the minimal significance level of reported coevolutionary interactions and controlling for unobservable data (see the ‘Overview’ section within the CoPAP web server for more details).

### Evolutionary model

The inference of coevolutionary interactions is dependent on ancestral mapping of gain and loss events along the tree. The accuracy of such mapping depends on the underlying evolutionary model (15). The simplest model assumes that a single evolutionary rate characterizes all characters and allows obtaining results in the shortest time. However, typically this model is extremely unrealistic, as different genes evolve in different rates. Thus, the default model allows for among-gene rate variation, by assuming that the rates are gamma distributed with an additional invariant category. A more advanced mixture model is additionally available, which allows both the gain rate and the loss rate to independently vary among genes (14). The free parameters of all evolutionary models are estimated using maximum likelihood from the data. Further details regarding all available parameters are provided in the ‘Overview’ section in the web server.

### A comparative platform for estimating performance of coevolution inference using simulations

Using simulations we evaluated the CoPAP methodology and compared it with the explicit models for pairwise coevolution as implemented in BayesTraits (31) and with a phylogeny-independent approach, based on correlation between observed (extant) patterns of presence and absence, which we term ‘Observed Correlation’ (19). We found area under precision-recall curve of 0.527, 0.453 and 0.292 for CoPAP, BayesTraits and ‘Observed Correlation’ methods, respectively. These results indicate that CoPAP infers coevolving characters more accurately than both other methods. Notably, CoPAP’s run time was <1% of that of BayesTraits but much higher than ‘Observed Correlation’. Further details are provided in the ‘Benchmark’ section within the CoPAP web server.

### Case study: the bacterial genes coevolutionary network

We used CoPAP to analyze 4258 bacterial clusters of orthologous genes (COGs) across 681 bacterial genomes. Phyletic patterns were retrieved from eggNOG (36) and the tree from Wu et al. (37). This is the first model-based coevolutionary analysis of such extensive data, substantially larger than the data previously analyzed with this method [282 species (32)], or a previous coevolutionary analysis based on the parsimony approach [163 species (29)].

**Figure 1.**
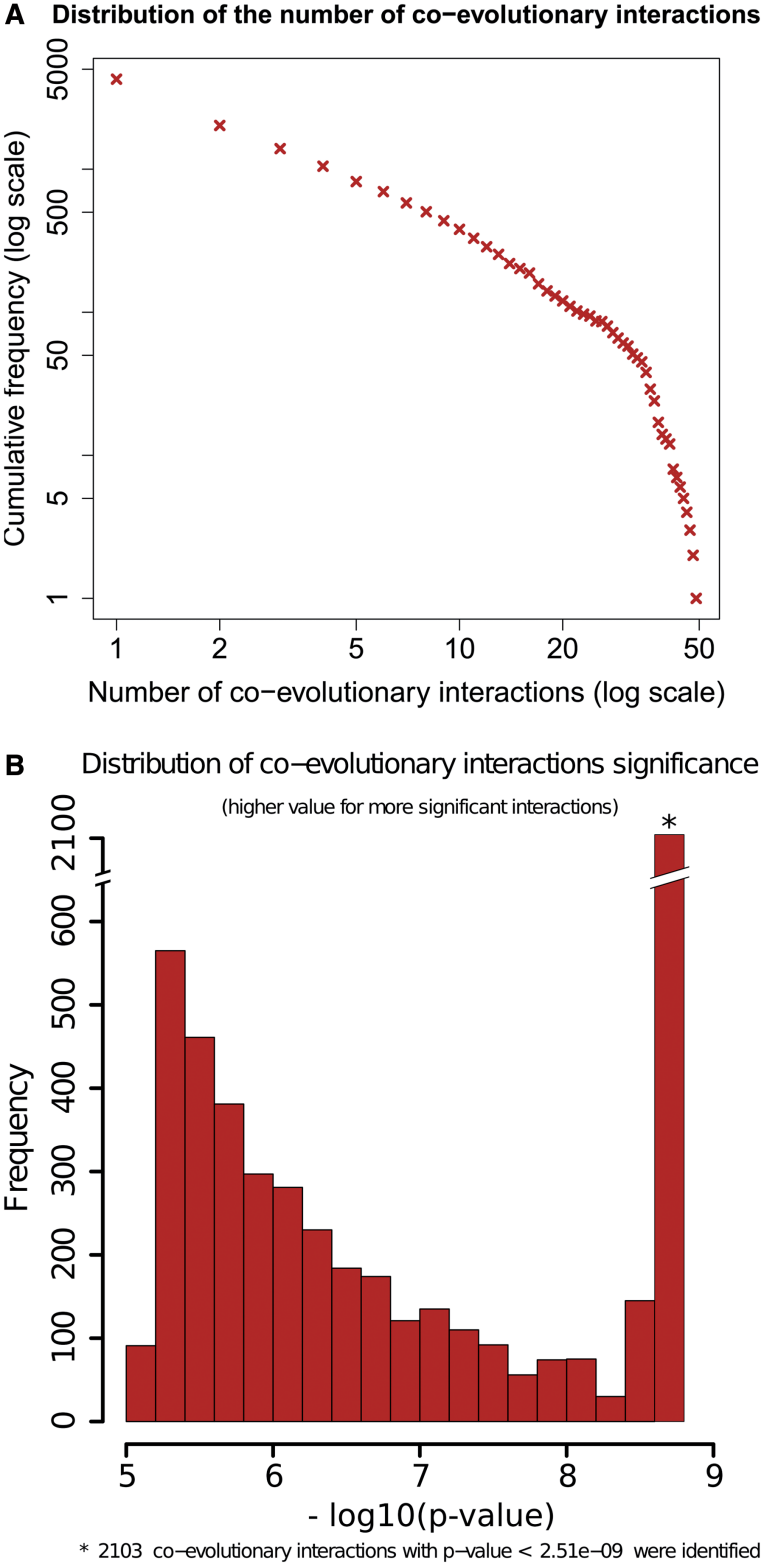
Global properties of the coevolutionary network. The global properties are illustrated with graphs that are automatically produced by CoPAP. (A) Distribution of the number of interactions. (B) Frequency of various significance levels of coevolutionary interactions. The high frequency for top interactions in this example is the result of limited number of simulations. Thus, all the strongest coevolutionary interactions fall in the top-significance bin with *P* < 2.51e-09.

CoPAP identified 5605 significant interactions (with a significance level of alpha = 0.01 and controlling for false discovery rate). Out of the 4258 COGs analyzed, almost 40% (1664) were found to be involved in strong coevolutionary interactions. CoPAP automatically produces graphical representation of the global properties of the coevolution network. Figure 1 includes examples of such graphical representations illustrating the distribution of the number of interactions (i.e. degree distribution among genes, Figure 1A), and the frequency of various significance levels of coevolutionary interactions (Figure 1B).

**Figure 2.**
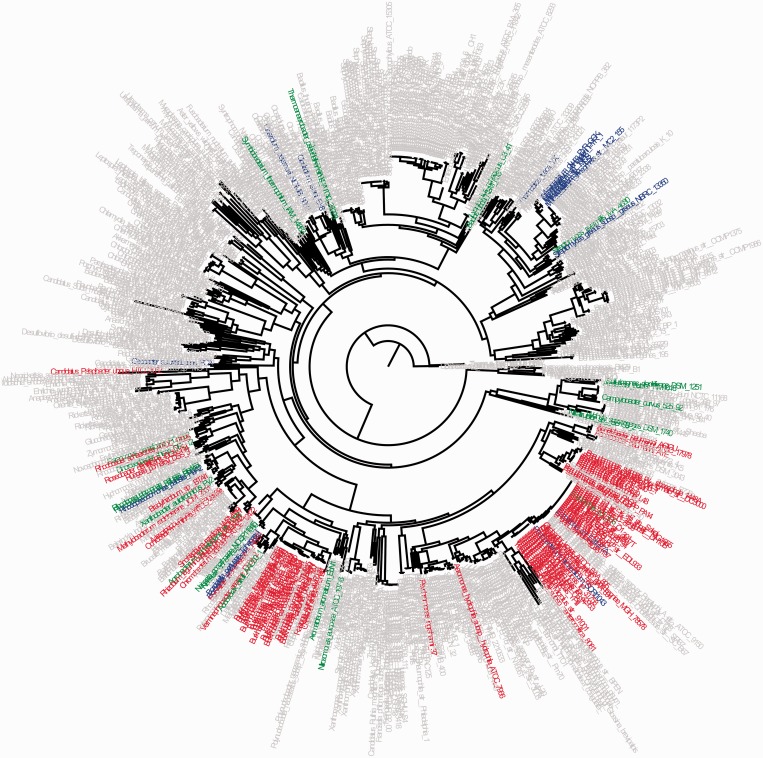
Projecting the phyletic patterns of two coevolving genes onto the tree. CoPAP allows automatic visualization of the presence–absence pattern for a given pair of genes. The pattern for a given pair is mapped onto the tree with taxa names colored according to presence in both (‘11’, red), absence in both (‘00’, gray), presence in the first only (‘10’, green) or presence in the second only (‘01’, blue). Here, the patterns of COG4521 (ABC-type taurine transport system, periplasmic component) and COG4525 (ABC-type taurine transport system, ATPase component) are presented. In this case, the high similarity in their phyletic patterns (as seen by the dominant red and gray colors) is in line with CoPAP’s inference of a statistically significant coevolution.

CoPAP allows users to easily inspect presence–absence patterns for genes of interest with respect to their underlying phylogeny using FigTree http://tree.bio.ed.ac.uk/software/figtree/ and Archaeopteryx (38). Figure 2 presents the patterns of two coevolving genes, COG4521 (ABC-type taurine transport system, periplasmic component) and COG4525 (ABC-type taurine transport system, ATPase component) using FigTree.

**Figure 3.**
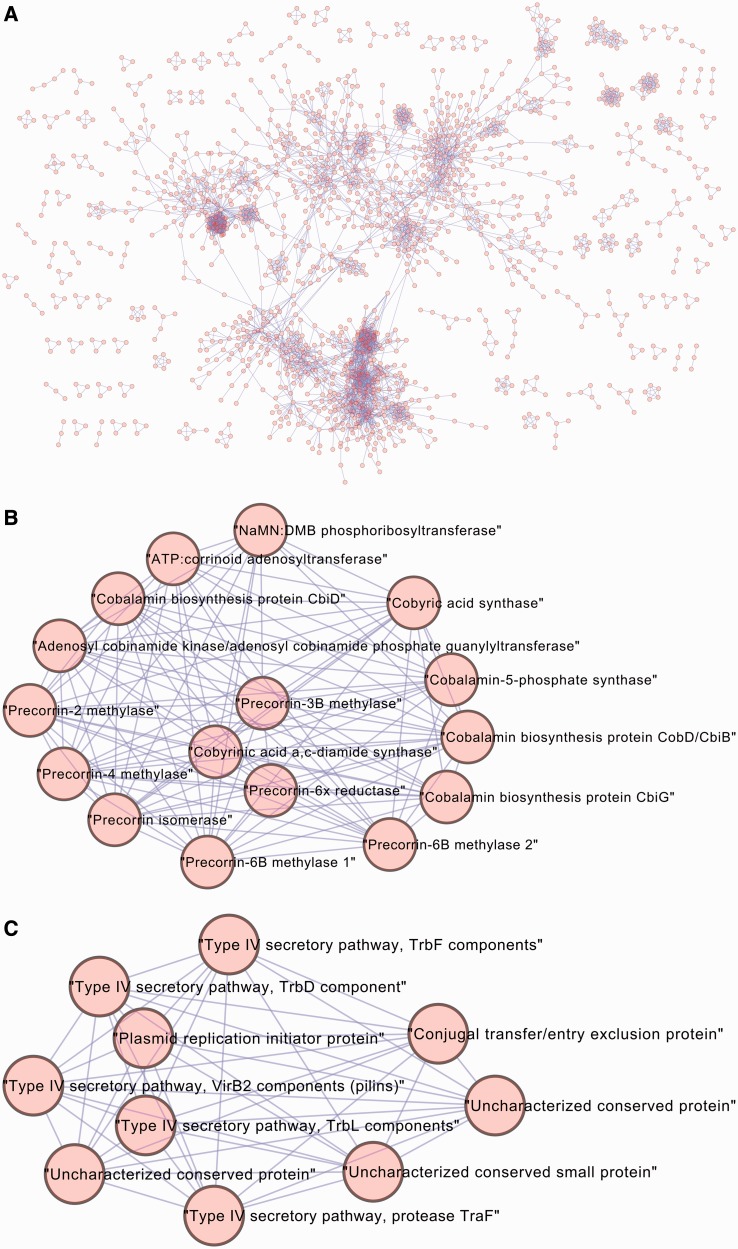
Visualization and analysis of the network using Cytoscape. The Cytoscape platform is deployed by CoPAP, allowing in-depth analysis of the coevolutionary network. (**A**) Global view of the bacterial coevolutionary network. Examples of clusters automatically detected using the transitivity clustering plug-in for Cytoscape: (**B**) B12 (Cobalamin) synthesis; (**C**) Type IV secretion/conjugation.

The reconstructed coevolutionary network is available for download as a detailed text file. Additionally, CoPAP provides advanced network visualization and analysis by automatically loading the network to the Cytoscape platform (33). Figure 3A exemplifies network visualization using Cytoscape for our case study. Cytoscape further allows many functions for network analysis. The detection of groups of genes that coevolve with each other is of special interest, as it may provide valuable insights revealing modularity within bacterial genomes. For this purpose, Cytoscape was preloaded with plug-ins to analyze clusters within the network. In our case study, we clustered genes using the transitivity clustering plug-in (34) to reveal hundreds of clusters of coevolving genes. Coevolving clusters of genes show overwhelming agreement with known function annotation: >90% of the 54 largest clusters (with at least five members) consist of genes with a similar function. A cluster is considered as consisting of genes with similar function if at least 80% of its members share a function, such as members of the same metabolic pathway (e.g. B12 Synthesis, Figure 3B), genes having a similar function description or biological process (e.g. Type IV secretion/conjugation, Figure 3C), genes that contribute to the same phenotype or trait (e.g. motility-related genes, see ‘Gallery’ section in the web server), genes encoding subunits of a protein complex (e.g. NADH:ubiquinone oxidoreductase complex, see ‘Gallery’) or genes sharing the same COG functional category (e.g. ‘amino acid transport and metabolism’, see ‘Gallery’). The inferred coevolving clusters represent functional modules in bacterial genomes.

## CONCLUSION

The observation that by-and-large clusters of coevolving genes are annotated with similar biological functions strongly supports the validity of this approach to extract meaningful biological interactions. This observation also suggests a crucial role for coevolutionary analysis in uncovering dependencies and associations between evolving genes. The publically available web server we present here is suitable for analyzing various binary-coded data and thus, has the potential to facilitate further biological understanding with the discovery of additional coevolutionary networks.

## References

[1] Mirkin BG, Fenner TI, Galperin MY, Koonin EV (2003). Algorithms for computing parsimonious evolutionary scenarios for genome evolution, the last universal common ancestor and dominance of horizontal gene transfer in the evolution of prokaryotes. BMC Evol. Biol..

[2] Hao W, Golding GB (2004). Patterns of bacterial gene movement. Mol. Biol. Evol..

[3] Cohen O, Rubinstein ND, Stern A, Gophna U, Pupko T (2008). A likelihood framework to analyse phyletic patterns. Philos. Trans. R. Soc. Lond. Ser. B Biol. Sci..

[4] Templeton AR (1983). Phylogenetic inference from restriction endonuclease cleavage site maps with particular reference to the evolution of humans and the apes. Evolution.

[5] Felsenstein J (1992). Phylogenies from restriction sites: a maximum-likelihood approach. Evolution.

[6] Nei M, Tajima F (1985). Evolutionary change of restriction cleavage sites and phylogenetic inference for man and apes. Mol. Biol. Evol..

[7] Simmons MP, Ochoterena H (2000). Gaps as characters in sequence-based phylogenetic analyses. Syst. Biol..

[8] Belinky F, Cohen O, Huchon D (2010). Large-scale parsimony analysis of metazoan indels in protein-coding genes. Mol. Biol. Evol..

[9] Csurös M (2006). On the estimation of intron evolution. PLoS Comput. Biol..

[10] Carmel L, Wolf YI, Rogozin IB, Koonin EV (2007). Three distinct modes of intron dynamics in the evolution of eukaryotes. Genome Res..

[11] Ronquist F (2004). Bayesian inference of character evolution. Trends Ecol. Evol..

[12] Hao W, Golding GB (2006). The fate of laterally transferred genes: life in the fast lane to adaptation or death. Genome Res..

[13] Spencer M, Sangaralingam A (2009). A phylogenetic mixture model for gene family loss in parasitic bacteria. Mol. Biol. Evol..

[14] Cohen O, Pupko T (2010). Inference and characterization of horizontally transferred gene families using stochastic mapping. Mol. Biol. Evol..

[15] Cohen O, Pupko T (2011). Inference of gain and loss events from phyletic patterns using stochastic mapping and maximum parsimony–a simulation study. Genome Biol. Evol..

[16] Dutheil J, Pupko T, Jean-Marie A, Galtier N (2005). A model-based approach for detecting coevolving positions in a molecule. Mol. Biol. Evol..

[17] Pollock DD, Taylor WR, Goldman N (1999). Coevolving protein residues: maximum likelihood identification and relationship to structure. J. Mol. Biol..

[18] Poon AF, Lewis FI, Pond SL, Frost SD (2007). An evolutionary-network model reveals stratified interactions in the V3 loop of the HIV-1 envelope. PLoS Comput. Biol..

[19] Glazko GV, Mushegian AR (2004). Detection of evolutionarily stable fragments of cellular pathways by hierarchical clustering of phyletic patterns. Genome Biol..

[20] Wu J, Kasif S, DeLisi C (2003). Identification of functional links between genes using phylogenetic profiles. Bioinformatics.

[21] Marcotte EM, Xenarios I, van Der Bliek AM, Eisenberg D (2000). Localizing proteins in the cell from their phylogenetic profiles. Proc. Natl Acad. Sci. USA.

[22] Dutkowski J, Tiuryn J (2009). Phylogeny-guided interaction mapping in seven eukaryotes. BMC Bioinformatics.

[23] Ettema T, van der Oost J, Huynen M (2001). Modularity in the gain and loss of genes: applications for function prediction. Trends Genet..

[24] Zhou Y, Wang R, Li L, Xia X, Sun Z (2006). Inferring functional linkages between proteins from evolutionary scenarios. J. Mol. Biol..

[25] Zheng Y, Roberts RJ, Kasif S (2002). Genomic functional annotation using co-evolution profiles of gene clusters. Genome Biol..

[26] Pellegrini M, Marcotte EM, Thompson MJ, Eisenberg D, Yeates TO (1999). Assigning protein functions by comparative genome analysis: protein phylogenetic profiles. Proc. Natl Acad. Sci. USA.

[27] Huynen MA, Snel B (2000). Gene and context: integrative approaches to genome analysis. Adv. Protein Chem..

[28] Pellegrini M (2012). Using phylogenetic profiles to predict functional relationships. Methods Mol. Biol..

[29] Cordero OX, Snel B, Hogeweg P (2008). Coevolution of gene families in prokaryotes. Genome Res..

[30] Campillos M, von Mering C, Jensen LJ, Bork P (2006). Identification and analysis of evolutionarily cohesive functional modules in protein networks. Genome Res..

[31] Barker D, Meade A, Pagel M (2007). Constrained models of evolution lead to improved prediction of functional linkage from correlated gain and loss of genes. Bioinformatics.

[32] Cohen O, Ashkenazy H, Burstein D, Pupko T (2012). Uncovering the co-evolutionary network among prokaryotic genes. Bioinformatics.

[33] Smoot ME, Ono K, Ruscheinski J, Wang PL, Ideker T (2011). Cytoscape 2.8: new features for data integration and network visualization. Bioinformatics.

[34] Wittkop T, Emig D, Truss A, Albrecht M, Bocker S, Baumbach J (2011). Comprehensive cluster analysis with transitivity clustering. Nat. Protoc..

[35] Saitou N, Nei M (1987). The neighbor-joining method: a new method for reconstructing phylogenetic trees. Mol. Biol. Evol..

[36] Powell S, Szklarczyk D, Trachana K, Roth A, Kuhn M, Muller J, Arnold R, Rattei T, Letunic I, Doerks T (2012). eggNOG v3.0: orthologous groups covering 1133 organisms at 41 different taxonomic ranges. Nucleic Acids Res..

[37] Wu D, Hugenholtz P, Mavromatis K, Pukall R, Dalin E, Ivanova NN, Kunin V, Goodwin L, Wu M, Tindall BJ (2009). A phylogeny-driven genomic encyclopaedia of Bacteria and Archaea. Nature.

[38] Han MV, Zmasek CM (2009). phyloXML: XML for evolutionary biology and comparative genomics. BMC Bioinformatics.

